# Comparative Study of Ocular Pharmacokinetics of Topical 0.3% Gatifloxacin Eye Gel and Solution in Rabbits

**DOI:** 10.3390/antibiotics11040502

**Published:** 2022-04-10

**Authors:** Manli Liu, Xin Zhao, Yao Yang, Qiang Yang, Jieting Zeng, Yujie Li, Xiaofeng Lin, Fang Duan

**Affiliations:** 1 State Key Laboratory of Ophthalmology, Zhongshan Ophthalmic Center, Sun Yat-sen University, Guangdong Provincial Key Laboratory of Ophthalmology and Visual Science, Guangdong Provincial Clinical Research Center for Ocular Diseases, Guangzhou 510060, China; liumanli1225@163.com (M.L.); yangyao22@163.com (Y.Y.); jyj057@126.com (J.Z.); liyujie@gzzoc.com (Y.L.); 2 Shenyang Xingqi Pharmaceutical Co., Ltd., 68 Sishui Street, Shenyang 110164, China; zhaoxin@sinqi.com (X.Z.); yangqiang@sinqi.com (Q.Y.)

**Keywords:** gatifloxacin, ocular penetration, ocular pharmacokinetics

## Abstract

Few articles have reported drug concentrations of different ophthalmic dosage forms in the ocular tissues. This study aimed to determine the ocular pharmacokinetics of gatifloxacin 0.3% eye gel (GTX-Gel) and gatifloxacin 0.3% eye solution (GTX-Sol) at different time intervals after topical instillation in rabbits. A total of 126 healthy New Zealand rabbits were included, of which six rabbits did not receive antibiotics (control group). The remaining rabbits were randomly divided into four groups. GTX-Gel and GTX-Sol (50 μL) were topically instilled every hour in groups A1 and B1, respectively, and every two hours in groups A2 and B2, respectively, for 12 h. Ocular tissues were collected 2, 4, 8, 12, and 24 h after administration. Gatifloxacin concentration was measured using high-performance liquid chromatography coupled with tandem mass spectrometry. The drug reached peak concentrations (C_max_) in all tissues at 8–12 h. With the same administration frequency, the C_max_ was higher with GTX-Gel than with GTX-Sol (*p* < 0.05). Except for the iris-ciliary body, other ocular tissues did not show significant difference (*p* > 0.05) in gatifloxacin concentration between either pair of groups. Gatifloxacin ophthalmic gel was found to attain significantly higher concentrations than the ophthalmic solution in ocular tissues.

## 1. Introduction

Ophthalmic drug forms have been one of the most important and widely developed pharmaceuticals for several years [1,2,3]. Bacterial keratitis and endophthalmitis are severe forms of ocular infections that can lead to irreversible visual loss if not treated properly and timely [4,5,6]. To prevent or treat ocular infections, topical antibiotics must penetrate the relevant tissues at concentrations sufficient to eradicate the causative organisms [7].

Topical fluoroquinolone antibiotics are widely used in clinical practice. Gatifloxacin is a fourth-generation topical fluoroquinolone. Studies have shown that gatifloxacin is more potent than second- and third-generation fluoroquinolones, suggesting that it is superior to these in treating and preventing ocular infections caused by gram-positive organisms and atypical mycobacteria [8]. Low bioavailability of drugs after optical instillation is observed because of the complicated anatomy of the eye, the small absorptive surface, lipophilicity of corneal epithelium, binding of the drug with the proteins in tear fluid, and defense mechanisms of the eye, such as tear formation, blinking, and flow of substances through the nasolacrimal duct [9,10,11]. Previous studies have demonstrated that prolonged precorneal residence and delay the elimination of the drug from the precorneal area can effectively increase the bioavailability of drugs [12]. To achieve the required drug concentration in the target tissue and sustain it for an appropriate period of time, polymers, such as carbomers, hyaluronic acid and hypromellose among others were introduced into formulations of conventional ophthalmic dosage forms. Carbomer as bioadhesive polymers can prolong the retention time of the drug on the surface of the eye and drug release to increase the ocular absorption [13]. The main advantages of carbomer include its excellent biocompatibility and mucoadhesiveness, which increased both the rate and extent of drug transfer from oil to the aqueous phase. Its use as a vehicle in ocular drug delivery has been reviewed previously [14].

Studies have revealed that carbomers were used in timolol liquid formulations to form gels to increase the viscosity of the formulation, which would, in turn, reduce the frequency of drug application [15]. In patients with keratitis and endophthalmitis, the antibiotics need to penetrate relevant tissues at sufficient concentrations. Thus, carbomers are added to gatifloxacin eye drops to increase its tissue permeability, and lower its dosing frequency for the patients’ convenience [16]. The purpose of this study was to compare the pharmacokinetic and distribution of gatifloxacin 0.3% ophthalmic gel (GTX-Gel) and gatifloxacin 0.3% ophthalmic solution (GTX-Sol) in the ocular tissues after topical application once every hour and every 2 h.

## 2. Results

### 2.1. Method Development

**Figure 1:**
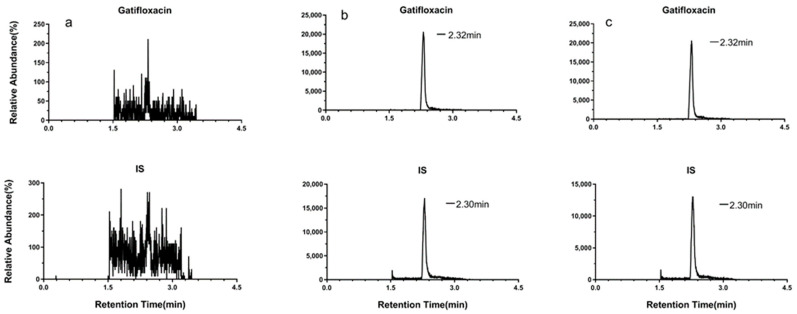
Representative multiple reaction monitoring (MRM) chromatograms of (**a**) a blank corneal homogenate; (**b**) a corneal homogenate spiked with 0.4 μg/mL gatifloxacin and the IS; and (**c**) a corneal sample from the rabbit in group A1.

The study method was validated for selectivity, linearity, accuracy, precision, matrix effect, recovery, and stability according to the US Food and Drug Administration bioanalytical method validation guidelines. In the chromatograms of plasma and ocular tissue homogenates from untreated rabbits, no endogenous interfering peaks were found at the retention times of gatifloxacin and Internal Standard (IS). Corneal homogenate was used as an example to show specificity, as shown in Figure 1.

The linearity of the method was studied by analyzing the calibration standards in duplicate at each concentration level in different biological specimens. All calibration curves showed good linearity and all correlation coefficients (r) were >0.99 (Table 1).

The results mentioned above and in Table 2 and Table 3 show that this method was successfully validated and could be applied to studies of ocular pharmacokinetics of gatifloxacin in rabbits.

### 2.2. Pharmacokinetic and Ocular Tissue Distribution Study

**Figure 2:**
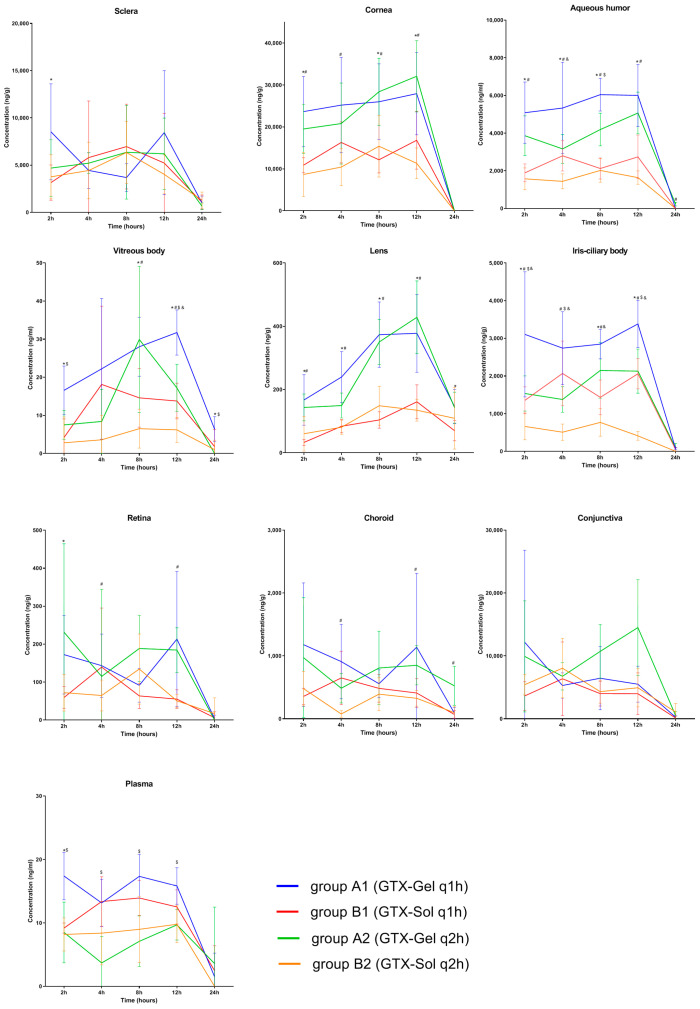
Concentration-time values of gatifloxacin in ocular tissues and plasma. Each point represents the mean concentration (*n* = 5–6). ±: the standard error of the mean. *: The difference was statistically significant between groups A1 and B1; #: The difference was statistically significant between groups A2 and B2; $: The difference was statistically significant between groups A1 and A2; &: The difference was statistically significant between groups B1 and B2 (*p* < 0.05).

The concentration-time values of gatifloxacin in ocular tissues and plasma are presented in Figure 2, and the estimated pharmacokinetic values are summarized in Table 4.

The drug concentration in the sclera and conjunctiva fluctuated at 4–10 μg/g in each period in the 4 gourps. No significant difference in the drug concentration was found between groups (*p* > 0.05). The drug concentration in the posterior segment of the eyeball was low, such as in the vitreous body, retina, and choroid. The value fluctuated between 0.01–1 μg/g. For the iris-ciliary body, gatifloxacin concentration was significantly higher in group A1 than other groups (*p* < 0.05).

After the administration of GTX-Gel or GTX-Sol every hour or every two hours for 12 h, the drug distribution in ocular tissues was ranked in the following order from highest to lowest: cornea > sclera > conjunctiva > aqueous humor > iris-ciliary body > choroid > lens > retina > vitreous body > plasma. The drug reached peak concentrations (C_max_) in the corneal tissue, aqueous humor, vitreous body, lens, iris-ciliary body, retina, and choroid at 8–12 h. C_max_ values of gatifloxacin in ocular tissues are summarized in Table 5.

With the same administration frequency, the between-group differences in C_max_ in the cornea, aqueous humor, vitreous body, lens, and iris-ciliary body were significant (*p* < 0.05). After the final dose, the gatifloxacin concentration in the cornea and aqueous humor was lower than the lower limit of quantification (20 mg/mL) in all groups. With the same administration frequency, between-group differences in the gatifloxacin concentration in the sclera, retina, choroid, conjunctiva, and plasma were not statistically significant (*p* > 0.05).

The mean area under curve during the 24 h period (AUC_0–24_) was significantly higher for GTX-Gel than for GTX-Sol in the ocular tissues (*p* < 0.05). In all groups, the lowest AUC_0–24_ was found in the plasma, which indicated that the drug was minimally absorbed into the systemic circulation. The mean plasma AUC_0–24_ was higher in the GTX-Gel groups than in the GTX-Sol groups, but the difference was not statistically significant (*p* > 0.05).

Except for the iris-ciliary body, no other tissues showed significant differences in gatifloxacin concentration between the groups A1 and A2, or between B1 and B2 (*p* > 0.05). These results indicate that when the administration frequency decreased from hourly to two-hourly, the concentration of gatifloxacin in the ocular tissues did not effectively decrease. It may be clinically significant to increase the administration frequency of 0.3% GTX-Gel or GTX-Sol to once every hour for the treatment of iris-ciliary body infections.

## 3. Discussion

The most important finding of this study was that 0.3% GTX-Gel has greater penetration of ocular tissues than GTX-Sol. When the administration frequency of either formulation decreased from once every hour to once every 2 h, the drug concentration in the ocular tissues did not effectively decrease.

Previous studies have shown that gatifloxacin is more potent than second- and third-generation fluoroquinolones [17]. Compared with older fluoroquinolones, gatifloxacin is characterized by higher in vitro activity against gram-positive bacteria, most notably *Streptococcus pneumoniae*. It also exhibits enhanced activity against atypical pathogens (*Chlamydia* and *Mycoplasma* species) and some anaerobic bacterial pathogens [8,18].

Perioperative antibiotic prophylaxis for endophthalmitis following eye surgery is the most effective form of prophylaxis [7]. Fluoroquinolones possess the advantages of broad antibiotic spectra, high efficiency, low toxicity, and high corneal penetration, and they exert antimicrobial effects by affecting the activities of DNA gyrase and topoisomerase IV [19,20]. Therefore, fluoroquinolone drops are one of the most common perioperative agents used in clinics [21]. That was the main reason we chose to study the pharmacokinetic and distribution of gatifloxacin. A previous study investigated the penetration of 0.3% gatifloxacin ophthalmic gel and 0.3% gatifloxacin ophthalmic solution into the aqueous humor after topical application in patients undergoing phacoemulsification procedures. They found that the bioavailability of ophthalmic gel was approximately 2-fold greater than that of the solution, which indicated that the gel maintained the longest time and had the best penetration and bioavailability [22].

The continuing escalation of antimicrobial-resistance indicates that slowing the rate at which resistance develops in pathogens is of paramount importance. The concept of mutant prevention concentration (MPC) represents a novel in vitro measurement of fluoroquinolone potency [23,24]. Tissue penetration by a topical antibiotic is important for attaining MPCs, as it affects the ability of the drug to achieve a concentration well above the minimum inhibitory concentrations (MIC) for a given microorganism. In essence, MPC defines the antimicrobial drug concentration threshold. Generally, a C_max_/MIC ratio of 8 to 10 has been associated with effective treatment. When comparing the gatifloxacin ocular tissue concentrations achieved in this study with MPCs derived from the MICs in the study conducted by Hosaka et al. [25], we found that GTX-Gel achieved MPCs of 8.92 for *Staphylococcus haemolyticus* (SH) and *Pseudomonas aeruginosa* (PA)in the cornea. This indicated that GTX-Gel would useful for PA keratitis in rabbit eye, which was confirmed by Mah F S et al. [26] in the previous study. Table 6 lists the in vitro data on MIC90 of gatifloxacin for these bacterial isolates.

However, GTX-Sol could not achieve MPCs for *S. haemolyticus* and *P. aeruginosa* in the cornea. Neither GTX-Gel nor GTX-Sol could achieve MPCs for these pathogens in the aqueous humor or iris-ciliary body. However, GTX-Gel could achieve the MPC for *S. pneumoniae* in the aqueous humor and iris-ciliary body. All MPC values were higher in the GTX-Gel groups than in the GTX-Sol groups. Notably, neither GTX-Gel nor GTX-Sol could achieve MPC or MIC in the vitreous body. This is consistent with other studies investigating topically administered drug penetration, which show that negligible concentrations were detected in the vitreous body [27,28]. This could be a major obstacle in the treatment of endophthalmitis.

Another important finding of the current study is that even with an hourly dosing for 12 h, the concentration of gatifloxacin in the ocular tissues was undetectable 12 h after the application of the last dose. This indicates that maintaining the drug concentration at required levels at night is difficult, which is a major problem in clinical practice.

Our results confirmed that, compared to GTX-Sol, GTX-Gel exhibited better penetration into the cornea, aqueous humor, and iris-ciliary body, and this indicates that patients treated with this formulation could demonstrate a lower tendency to develop resistant organisms.

GTX-Gel contains gatifloxacin (0.3%), carbomer, hyaluronic acid and other excipients. These substances increase viscosity of GTX-Gel to 2 Pa s. The value of viscosity around 5–6 Pa s was reported to obtain the highest mucoadhesiveness of the polymeric dispersions [29]. Although CTX-Gel does not achieve the best viscosity, but it provides optimal comfort and does not blur the vision. The increased viscosity makes the drug enable longer contact with the eye surface, and thus improve the bioavailability of the formulation [30]. These ingredients have protective effects on the cornea [1,31,32]. These findings suggest that 0.3% GTX-Gel could promote corneal wound healing and that it requires a low frequency of drug application. In addition, they exhibit better physicochemical characteristics, such as bioadhesion and ocular tolerance, compared to conventional ophthalmic preparations, as a consequence of prolonged pre-corneal residence time. Furthermore, as ophthalmic gels do not cause “blurred vision”, they can be comfortably applied and, thus, are more acceptable for patients [22]. The formula has been granted an invention patent of China National Intellectual Property Administration (CNIPA) (Patent Number: CN102078284B) and a United States Patent (Patent Number: US8901131B2) [33].

The strength of this study includes the demonstration of differences in drug concentrations in all ocular tissues. The limitations of this study include the small sample size and the short duration of the experiment. We did not detect the duration of the peak concentration of either of the dosage forms.

In conclusion, this study demonstrated that, compared with topically administered 0.3% GTX-Sol, 0.3% GTX-Gel showed greater penetration into the cornea, aqueous humor, vitreous body, lens, and iris-ciliary body after administration every hour or two hours for 12 h. Thus, 0.3% GTX-Gel exhibited an advantage over 0.3% GTX-Sol, as it requires a lower application frequency because of better penetration into the ocular tissues. In addition, when the frequency of GTX-Gel or GTX-Sol decreased from once every hour to once every 2 h, the drug concentration in the ocular tissues did not effectively decrease. We believe that our results will provide guidance for the clinical use of these medications. GTX-Gel offers a more effective ophthalmic therapy for ocular infections than conventional eye drops.

## 4. Materials and Methods

### 4.1. Materials

One hundred and twenty-six healthy New Zealand white rabbits (63 males and 63 females), ranging from 2.0 to 2.5 kg in weight were raised for at least 1 week under standardized temperature, humidity, and lighting conditions before the experiment. All animals were treated in accordance with the guidelines of the Association for Research in Vision and Ophthalmology Statement for the Use of Animals in Ophthalmic and Vision Research. Ethics approval for the study protocol was obtained from the appropriate institutional review board.

### 4.2. Preparation of Calibration Standards and Quality Control Samples

Standard stock solutions of gatifloxacin and the IS were prepared separately in methanol at a concentration of 1.0 μg/μL. Stock gatifloxacin solutions were diluted with methanol to obtain a series of working solutions (0.4–4 μg/mL). The concentration of the IS working solution was 0.4 μg/mL. Quality control working solutions at concentrations of 0.8, 1, and 3.2 μg/mL were prepared from a separately prepared 1000 μg/mL stock solution of gatifloxacin. All the solutions were stored at 4 °C until further analysis.

Calibration standards were freshly prepared by placing 25 μL of each gatifloxacin working solution into 50 μL of blank rabbit plasma or ocular tissue homogenates, added with 25 μL of the IS working solution and 175 μL of methanol. The mixture was vortex-mixed for 1 min and centrifuged at 14,500 rpm for 10 min. Then, 40 μL of the supernatant, containing 16 × 10^−5^ L of methanol, was vortex-mixed at 14,500 rpm for 5 min. An aliquot of 7 × 10^−6^ L of the supernatant was injected into a liquid chromatography with tandem mass spectrometry (LC/MS/MS) system. Quality control samples were also prepared using the method described above with final concentrations of 40,500, and 1600 ng/mL.

### 4.3. Sample Processing Methods

The animals were randomly divided into five groups. Six rabbits in the control group did not receive any antibiotics, and the remaining 120 rabbits were randomly divided into four groups; 5 × 10^−5^ L of GTX-Gel (group A1) or GTX-Sol (group B1) was instilled in the conjunctival sac of the left eye every hour min for 12 h. Similarly, 50 μL of GTX-Gel (group A2) or GTX-Sol (group B2) was instilled every 2 h for 12 h. GTX-Gel and GTX-Sol (0.3%, for research only) were obtained from Shenyang Xingqi Pharmaceutical Co., Ltd. (Shenyang, China). Approximately 2 mL of blood was collected from the heart of each rabbit at 2, 4, 8, 12, and 24 h after topical instillation. The blood samples were poured into a tube containing heparin and mixed thoroughly. The rabbits were euthanized by an overdose of anesthesia, and the aqueous humor, conjunctiva, cornea, iris-ciliary body, lens, vitreous body, retina, choroid, and sclera were collected from the left eye. The aqueous humor and vitreous body were collected by aspiration with syringes. The solid tissues, except for the retina and choroid, were procured and rinsed briefly with physiological saline solution to remove blood, blotted on filter paper, and then weighed. All specimens were stored at −80 °C until analysis. Samples at each time interval were collected half an hour after the dose was administered. The sampling times were within a window of ±5 min from the assigned time.

Solid ocular tissues were individually homogenized in double-distilled water at 1:10 (*w/v*), except for the retina, choroid, and lens (for which 1:3 *w/v* was used). The plasma, aqueous humor, and vitreous humor previously collected from the treated rabbit eye were used as samples directly. Next, 50 μL of the diluted solution was transferred to an Eppendorf tube^®^, and 25 μL of the IS working solution and 175 μL of methanol were added. The mixture was then treated using the same procedure followed for previous samples. The gatifloxacin concentrations in the solid ocular tissues were calculated by dividing the total amount of extracted drug in each specimen by the tissue weight.

### 4.4. Pharmacokinetic and Statistical Analysis

Drug concentrations in the corneal tissues and aqueous humor were determined using the previously described HPLC-MS/MS method. The C_max_ was obtained from visual inspection of the data, and the AUC was calculated using the linear trapezoidal rule, as described by Nedelman et al. [34] Statistical analysis was performed using Student’s two-tailed t test, and by analysis of variance and Scheffe’s test. 

## 5. Conclusions

This study demonstrated that, compared with topically administered 0.3% GTX-Sol, 0.3% GTX-Gel showed greater penetration into the ocular tissues after administration. When the frequency of GTX-Gel or GTX-Sol decreased from once every hour to once every 2 h, an equivalent gatifloxacin concentration could be achieved in the ocular tissues. The use of topically administered GTX-Gel or GTX-Sol could not achieve an effective antimicrobial drug concentration in the vitreous body, furthermore, the concentration of gatifloxacin in the ocular tissues was undetectable when the medication was stopped at night for 12 h.

## Figures and Tables

**Table 1:** Calibration curves for the determination of gatifloxacin concentration in different biological specimens.

Biological Specimens	Slope	Intercept	Correlation Coefficient	Regression Equation
Sclera	0.0053	0.004000	0.992	*y* = 0.00529x + 0.004
Cornea	0.00632	0.018600	0.991	*y* = 0.00632x + 0.0186
Aqueous humor	0.00524	0.004550	0.994	*y* = 0.00524x + 0.00455
Vitreous body	0.00418	0.004470	0.994	*y* = 0.00418x + 0.00447
Lens	0.00743	0.011800	0.994	*y* = 0.00743x + 0.0118
Iris-ciliary body	0.00414	0.006240	0.991	*y* = 0.00414x + 0.00624
Retina	0.00515	0.016800	0.992	*y* = 0.00515x + 0.0168
Choroid	0.00428	0.008830	0.991	*y* = 0.00428x + 0.00883
Conjunctiva	0.00768	0.002330	0.991	*y* = 0.00768x + 0.00233
Plasma	0.00415	0.007870	0.989	*y* = 0.00415x + 0.00787

**Table 2:** Precision, accuracy, recovery, and matrix effect of gatifloxacin in the cornea (*n* = 6).

Biological Specimen	Nominal Concentration (ng/mL)	Measured Concentration (ng/mL)	Intraday (RSD %)	Interday (RSD %)	RE (%)	Recovery (%)	Matrix Effect (%)
cornea	40	37.6	5.8	6.9	−5.9	96.3 ±3.6	89.9 ± 3.2
	500	462.3	2.9	4.7	−7.5	94.5 ± 5.2	95.6± 6.3
	1600	1530.6	7.1	6.4	−4.3	101.5 ± 1.9	93.8 ± 9.6
IS						97.8 ± 14.4	89.6 ± 7.9

RSD: relative standard deviation, RE: relative error.

**Table 3:** Stability of gatifloxacin in the cornea (*n* = 3).

Nominal Concentration (ng/mL)	Postpreparative Stability		Freeze-Thaw Stability		Long-Term Stability		Short-Term Stability	
	RE %	RSD %	RE %	RSD %	RE %	RSD %	RE %	RSD %
40	−8.2	1.9	−5.7	6.9	−8.2	1.9	−0.8	5.2
1600	−2.1	4.5	−6.0	2.3	−2.1	4.5	2.7	3.1

**Table 4:** AUC_0–24_ values of gatifloxacin in ocular tissues and plasma.

Biological Specimens	AUC_0–24_ (μg·h/g or μg·h/mL)			
	Group A1	Group A2	Group B1	Group B2
Sclera	118.63	142.36	99.64	94.06
Cornea	450.36	471.62	253.87	205.69
Aqueous humor	98.38	75.93	42.59	28.68
Vitreous body	0.50	0.30	0.24	0.10
Lens	6.45	8.56	2.44	2.69
Iris-ciliary body	53.29	33.34	31.42	9.21
Retina	2.87	3.04	1.27	1.38
Choroid	16.98	16.59	8.31	5.96
Conjunctiva	111.60	226.50	75.09	98.28
Plasma	0.28	0.16	0.23	0.16

AUC: area under curve. AUC_0–24_ was determined by the area under the concentration-time curve from 0 to 24 h in ocular tissues and plasma samples after ocular administration of gatifloxacin in rabbits.

**Table 5:** C_max_ values of gatifloxacin in ocular tissues.

Biological Specimens	Group A1	Group A2	Group B1	Group B2
Sclera (μg/g)	8.52	6.36	6.96	6.34
Cornea (μg/g)	27.93	32.08	16.82	15.40
Aqueous humor (μg/mL)	6.00	5.07	2.74	2.02
Vitreous body (μg/mL)	0.03	0.03	0.01	0.01
Lens (μg/g)	0.38	0.43	0.16	0.15
Iris-ciliary body (μg/g)	3.38	2.15	2.06	0.77
Retina (μg/g)	0.21	0.23	0.14	0.13
Choroid (μg/g)	1.18	0.97	0.65	0.48
Conjunctiva (μg/g)	12.18	14.51	6.36	8.05

**Table 6:** In vitro activity of gatifloxacin against clinical isolates [16].

Organism (No. of Isolates)	MIC (µg/mL)	
	Range	90%
*Staphylococcus aureus* (34)	0.05–0.20	0.1
*Methicillin-resistant Staphylococcus aureus* (30)	0.05–0.20	0.2
*Staphylococcus epidermidis* (26)	0.05–0.39	0.2
*Staphylococcus haemolyticus* (25)	0.01–6.25	3.13
*Staphylococcus pneumoniae* (15)	0.20–0.39	0.39
*Escherichia coli* (26)	0.0125–0.05	0.05
*Pseudomonas aeruginosa* (35)	0.78–12.5	3.13

## Data Availability

The data presented in this study are available on request from the corresponding author.
